# Age-Related Changes in Human Peripheral Blood *IGH* Repertoire Following Vaccination

**DOI:** 10.3389/fimmu.2012.00193

**Published:** 2012-07-09

**Authors:** Yu-Chang Bryan Wu, David Kipling, Deborah K. Dunn-Walters

**Affiliations:** ^1^Department of Immunobiology, King’s College London School of MedicineLondon, UK; ^2^Institute of Cancer and Genetics, School of Medicine, Cardiff UniversityCardiff, UK

**Keywords:** B cell repertoire, aging, vaccine, immunoglobulin, IgA

## Abstract

Immune protection against pulmonary infections, such as seasonal flu and invasive pneumonia, is severely attenuated with age, and vaccination regimes for the elderly people often fail to elicit effective immune response. We have previously shown that influenza and pneumococcal vaccine responses in the older population are significantly impaired in terms of serum antibody production, and have shown repertoire differences by CDR-H3 spectratype analysis. Here we report a detailed analysis of the B cell repertoire in response to vaccine, including a breakdown of sequences by class and subclass. Clustering analysis of high-throughput sequencing data enables us to visualize the response in terms of expansions of clonotypes, changes in CDR-H3 characteristics, and somatic hypermutation as well as identifying the commonly used *IGH* genes. We have highlighted a number of significant age-related changes in the B cell repertoire. Interestingly, in light of the fact that IgG is the most prevalent serum antibody and the most widely used as a correlate of protection, the most striking age-related differences are in the IgA response, with defects also seen in the IgM repertoire. In addition there is a skewing toward IgG2 in the IgG sequences of the older samples at all time points. This analysis illustrates the importance of antibody classes other than IgG and has highlighted a number of areas for future consideration in vaccine studies of the elderly.

## Introduction

Until recently the methods available to study B cell repertoire were limited by the fact that the diversity of the repertoire was far greater than the number of sequences that could feasibly be studied. A random sampling of cells responding to vaccine would pick up the most prevalent Ig genes but would not give any indication of the diversity of cells responding to challenge. In mice the T-dependent NP response is a widely used and versatile tool for the study of immune responses, it tends to be restricted to use of the V186.2 heavy chain. In humans the isolation of rearranged Ig genes with more than one type of *IGHV* gene has indicated that there might be diversity in the response, but the numbers of sequences studied in these experiments have been low. One of the best examples of repertoire analysis was by Kolibab et al. (2005a) who looked at approximately 1300 sequences from 40 different donors after immunization by the pneumococcal vaccine and identified the major *IGHV* genes in use. With the advent of high-throughput sequencing methods we can now study the human immune response in much more detail.

A perennial problem in vaccination is that of vaccine inefficiency in older people. Vaccine-specific antibodies in older people are quantitatively and qualitatively impaired. For example, anti-pneumococcal polysaccharide (PPS) antibodies have lower opsonophagocytic index and affinity in the elderly than in healthy young adults (Kolibab et al., 2005b; Park and Nahm, 2011). *Streptococcus pneumoniae* infection is a serious complication secondary to influenza, and together these two diseases comprise a substantial infectious burden for children aged under two, the elderly and immunocompromised individuals (McCullers, 2006). In the UK and many other countries, co-administration of trivalent-influenza and pneumococcal vaccines has been recommended as the routine immunization schedule to protect these at-risk groups. Although influenza vaccines appear effective in most groups with nearly 70% vaccine efficacy (Osterholm et al., 2012), less than half of the older adults are protected by influenza vaccines (Nichol et al., 2007). While the immunogenicity of PPS vaccines in the protein-conjugated form (PCV) is much improved for young children, neither PCV nor 23-valent PPS vaccine (PPV-23) is able to confer an effective protection in the older population (Baxendale et al., 2010).

We have previously seen that there may be changes in the selection process during affinity maturation of B cells (Banerjee et al., 2002), and also that the B cell repertoire is often less diverse in old age with evidence of non-pathogenic clonal expansions (Gibson et al., 2009). This loss of diversity correlated with the health of the individual. Further investigation as to whether loss of diversity in the B cell repertoire correlated with poor vaccine responses against influenza and pneumonia indicated that it may be a contributory factor, but that other factors were likely also involved (Ademokun et al., 2011). These two vaccines generate different responses; influenza is believed to mainly induce IgG1/IgG3/IgA1 T-dependent responses (Brown et al., 1985; Hocart et al., 1990; Powers, 1994), while pneumococcal responses are thought to be T-independent and can lead to significant elevation of IgA2/IgG2 antibodies in the serum and mucosa (Lue et al., 1988; Carson et al., 1995; Sanal et al., 1999; Simell et al., 2006; Benckert et al., 2011).

To investigate the diversity of the response to these vaccines in more detail we have analyzed high-throughput sequencing data in order to characterize the response with respect to Ig gene usage and hypermutation whilst paying particular attention to the subclasses of antibodies involved. There are significant differences in the older vaccine response with respect to class and subclass of antibody, extent and timing of clonal expansions and focusing of the repertoire toward Ig sequences with higher mutation and shorter CDR-H3 regions.

## Materials and Methods

### Volunteers and sample collection

Six young (aged 19–45) and six older (aged 70–89) healthy volunteers were recruited as a part of the 2009/10 influenza vaccination program in Lambeth Walk GP Practice. Blood and serum samples were collected after obtaining written consent as approved by the Guy’s Hospital Research ethics committee, prior to vaccination at day 0 (D0) with the influenza (Influvac; Solvay, Southampton, UK), and 23-valent pneumococcal (Pneumovax II; Sanofi Pasteur MSD, Maidenhead, UK) vaccines and post vaccination at day 7 (D7) and day 28 (D28). PBMCs were isolated using Ficoll-Paque Plus (GE Healthcare, Buckinghamshire, UK) in conjunction with Leucosep tubes (Greiner Bio-One Ltd., Gloucestershire, UK), according to manufacturer’s instructions.

### Total RNA extraction and cDNA conversion

Total RNA was extracted from 2 × 10^7^ PBMCs per donor per time point using the RNeasy Mini Kit (Qiagen, UK). The SuperScript III First-Strand cDNA Synthesis System (Invitrogen, UK) was then used to convert RNA into cDNA according to the manufacturer’s protocol. In brief, a 200-μL cDNA reaction mix contained extracted total RNA, 500 ng Oligo(dT)_20_, 500 μM dNTPs, 400 U RNaseOut, 10 mM DTT, and 2000 U SuperScript III RT in 1× First-Stand RT buffer. The cDNA reaction was carried out as follows: 65°C for (5 min), 4°C (60 s); 50°C (1 h); 70°C (15 min).

### High-throughput sequencing of *IgH* genes

Ig genes were isolated by semi-nested, isotype-specific PCR reactions, as previously reported (Wu et al., 2010). Briefly, a 25-μL PCR1 reaction mix contained 6.25 μL of cDNA, 0.625 U Phusion DNA polymerase (NEB, UK), 200 μM each dNTP, 41.75 nM each of 6 *IGHV* gene family primers in conjunction with 250 nM of either IgM, pan-IgA, or pan-IgG constant region primers. Two microliters of PCR1 products were subsequently re-amplified using multiplex identifier (MID)-containing primers in a semi-nested reaction consisting of 0.5 U Phusion DNA polymerase, 200 μM each dNTPs, 41.75 nM each of the *IGHV* gene family/MID primers, and 250 nM of the nested constant region/MID primers in a 20-μL reaction volume. PCR conditions are as follows: 98°C for (30 s), 15 (PCR1), or 20 (PCR2) cycles of 98°C (10 s); 58°C (15 s); 72°C (45 s), and 1 cycle of 72°C (10 min). In order to produce sufficient quantity for high-throughput sequencing on the GS FLX System (Roche, Germany), eight different PCR1 for each sample, followed by 16 PCR2 (two per initial PCR1 round) reactions were performed for each isotype. This total sampling of cDNA was approximately equivalent to that from 2 × 10^5^ B cells. The downstream preparation of PCR products and data processing are as previously published (Wu et al., 2010).

### Detection of anti-PPS IgA antibodies

Serum anti-PPS IgA antibodies from immunized young (age 18–49, *n* = 39) and older (age 65–89, *n* = 27) healthy volunteers were measured by ELISA, as previous reported (Ademokun et al., 2011). In brief, the 89-SF standard (Bethesda, MD, USA) or serum samples were pre-absorbed with 10 μg/mL cell wall polysaccharide (CPS; Statens Serum Institute, Copenhagen, Denmark) or 10 μg/mL CPS in conjunction with 10 μg/mL 22F polysaccharide, respectively, before being serial diluted and incubated with microtiter plates coated with 100 μL per well of a combination of seven polysaccharide serotypes (4, 6B, 9, 14, 18C, 19F, and 23F, 1 μg/mL each; ATCC, Rockville, MD, USA) overnight at 4°C. After washing with TBS containing 0.1% Brij 35 (Sigma-Aldrich, St. Louis, MO, USA), HRP-conjugated goat anti-human IgA (Invitrogen) diluted at 1/4000 in PBS with 0.05% Tween-20 was added to the plates and incubated for 2 h, followed by another 2 h of incubation with TMB chromogen substrate (Invitrogen) in diethanolamine buffer, before terminating the reaction with 3 M NaOH. Serum anti-PPS IgA levels were read at OD 450 nM on an ELISA microplate reader and then compared with the 89-SF standard.

### Sequence analysis

Ig gene usage and CDR-H3 junction regions between the conserved first (cysteine) and last amino acid (tryptophan) were determined using IMGT V-QUEST (Wilkins et al., 1999). Internal isotype motifs in the constant regions of each sequence further identified subclasses of sequences. ProtParam was used to determine the physicochemical properties of the CDR-H3 peptide (Brochet et al., 2008). The grand average of hydropathicity (GRAVY) and aliphatic indices are positive indicators for peptide hydrophobicity and structural thermostability respectively (Ikai, 1980; Wilkins et al., 1999). The percentage match of each IGHV sequence to germline gene was returned by IMGT V-QUEST, and the level of hypermutation was calculated to be the percentage difference from the corresponding germline gene.

The DNA sequences of the CDR-H3 were used for clonotype clustering by a distance-matrix between all pairwise comparisons, as previously reported (Ademokun et al., 2011). When necessary, clonally related IGH sequences were aligned with putative germline genes and edited to remove homopolymer tract errors using DNAstar software (Laser Gene). Mutational phylogenetic trees of the edited IGH sequences were constructed by the multiple sequence alignment modes (MUSCLE 3.7) using the Phylogeny Analysis program (Dereeper et al., 2010).

### Statistics

Statistics were performed with GraphPad Prism 5.0. Most statistical analyses were one-way or repeated measures ANOVA (with Bonferroni post-test) and Kruskal–Wallis comparisons (with Dunn’s post-test). Wherever necessary, Chi-squared test (with Bonferroni post-test), paired *t*-test, and Mann–Whitney *U*-test was performed. To test association between different metrics, Pearson correlation analysis in conjunction with linear regression was performed.

## Results

### *IGH* repertoire changes in response to vaccination

**Figure 1:**
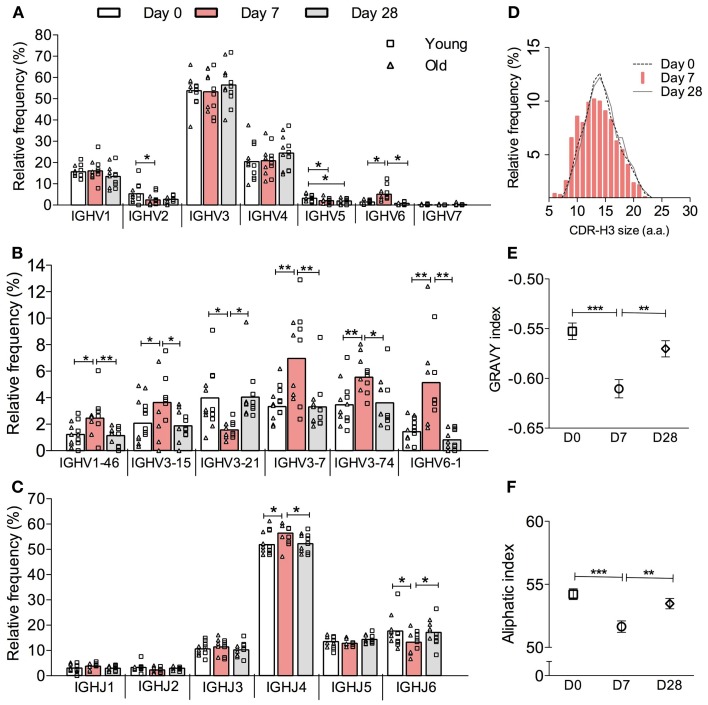
**Vaccine-induced changes in the overall *IGH* clonotype repertoire**. *IGH* genes in each sequence were analyzed by IMGT V-QUEST and then clustered into clones by CDR-H3 DNA sequence similarity. Only one sequence example per clone was chosen to represent its clonotype. The relative frequency (*y*-axis) of all clonotypes (IgA, IgG, and IgM combined) by **(A)** *IGHV* gene family, **(B)** individual *IGHV* gene, and **(C)** *IGHJ* gene usage (*x*-axis) was calculated for six young (squares) and six old (triangles) donors individually before being grouped as a whole cohort of 12 donors for repeated measures ANOVA comparison between D0 (open bars), D7 (red bars) and D28 (grey bars). Bars indicate MEAN. **(D)** CDR-H3 virtual spectratypes, showing the relative frequency (*y*-axis) of a particular CDR-H3 size (*x*-axis in amino acid numbers) within the whole CDR-H3 repertoire, was calculated using all clonotypes from 12 donors at D0 (dotted line), D7 (red bars), and D28 (solid line). The GRAVY and aliphatic indices for each CDR-H3 peptide was determined by ProtParam. The overall GRAVY **(E)** and aliphatic **(F)** indices all clonotypes from 12 donors were compared by Kruskal–Wallis comparisons with Dunn’s post-test between D0 (squares), D7 (circles), and D28 (diamonds). Error bars indicate ±SEM. *p < 0.05, **p < 0.005, and ***p < 0.0005.

High-throughput sequencing of samples from peripheral blood B cells in six young and six old donors in the course of vaccination produced 45,784 *IGH* sequences, which were grouped into 17,962 different clonotypes (i.e., a representative clone for that particular Ig gene rearrangement, Table 1). In order to investigate the effect of vaccination on the underlying repertoire without the influence of *in vivo* and *in vitro* clonal expansion, only clonotypes were included for this analysis. In general, the repertoire displayed resilience in that at D28 post vaccination it showed similar characteristics to D0 (before vaccination) despite significant changes at day 7 post vaccination. These changes at D7 included a change in expression of some *IGHV* and *IGHJ* genes such that significant differences were seen in IGH gene family usage (Figure 1A). Most notably, at D7 after vaccination there is a significant increase in the proportion of clonotypes that use *IGHV6-1*, *IGHV1-46*, and several *IGHV3* genes with a decrease in those using *IGHV2*, *IGHV4* gene families, and *IGHV3*-21 (Figure 1B). An increased usage of the shorter *IGHJ4* genes by 4.5% with a concomitant decrease in use of the longer *IGHJ6* gene (Figure 1C; *p* < 0.05) at D7 may explain the overall reduction in the CDR-H3 size by 2 nt (Figure 1D; *p* < 0.0005, Kruskal–Wallis test). The D7 population also has a more hydrophilic CDR-H3, as shown by a more negative GRAVY index (Figure 1E; *p* < 0.0005), and is also less aliphatic (Figure 1F; *p* < 0.0005).

**Table 1:** **Numbers of *IGH* sequence and clonotypes produced by high-throughput sequencing**.

Ages	Days	IgA	IgG	IgM	Unknown^3^	Total
Young (6 donors)	D0	886^1^/1424^2^	1035/1651	847/1208	413/737	3181/5020
	D7	976/5922	717/2707	641/1214	4912750	2825/12593
	D28	1070/1777	1298/2085	1036/1280	626/1059	4030/6201
Old (6 donors)	D0	928/1747	675/1528	809/1109	474/1175	2886/5559
	D7	644/1994	988/3801	579/998	418/1825	2629/8618
	D28	1223/5099	475/901	322/400	391/1393	2411/7793

*^1^The numbers of clonotypes refer to unique sequences, where only one example of a clonal expansion is counted after CDR-H3 clonotype clustering*.

*^2^The numbers of *IGH* sequences, generated from approximately 2 × 10^5^ B cells per sample, represent those that passed quality control with full immunoglobulin VDJ gene rearrangement*.

*^3^Unknown isotype refers to sequences that do not extend far enough to the constant region for isotype identification*.

### Age-related differences in vaccine-induced clonal expansion

**Figure 2:**
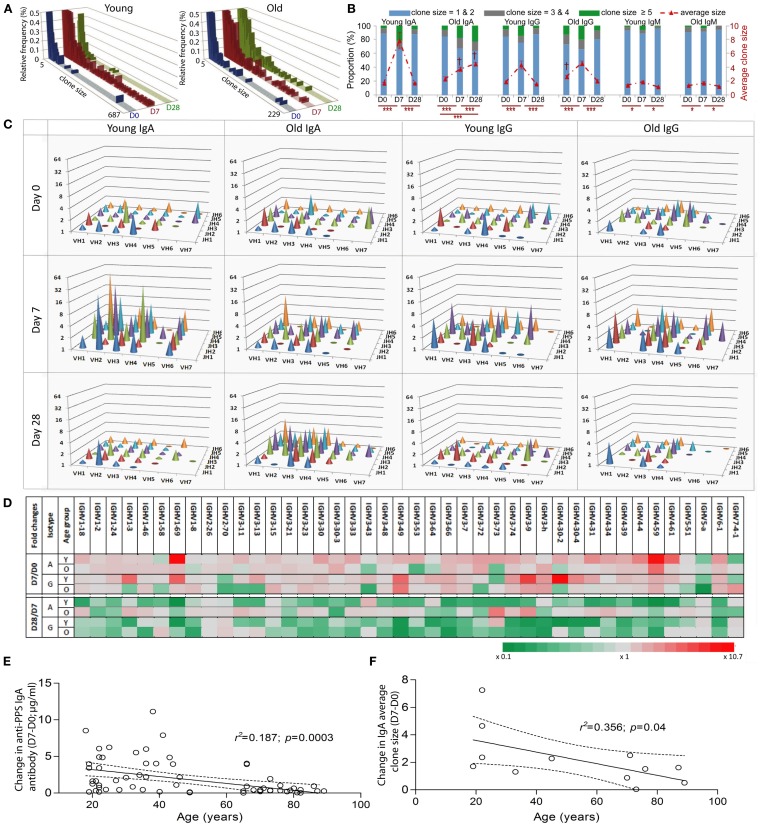
**Age-related differences in clonal expansion and serum anti-PPS IgA antibodies after vaccination**. The size of each clone was determined. **(A)** The relative proportion (*y*-axis; %) of clones of different sizes (*x*-axis) was calculated using a collection of total clonotypes (IgA + IgG + IgM) from six young and six old donors at D0 (blue bars), D7 (red bars), and D28 (green bars). **(B)** Clonotypes were grouped by isotype and size (clone size = 1 and 2 in blue bars, 3 and 4 in grey bars; 5 and above in green bars; Left *y*-axis in %), and the proportion of each group was calculated for six young and six old donors at D0, D7, and D28. The average clone size (red line; Right *y*-axis) was calculated for each donor individually to be the ratio of total sequence numbers to total clonotype numbers, before being compared between different days (*p < 0.05 and ***p < 0.0005; repeated measures ANOVA comparison) or between ages (**^†^p** < 0.005; one-way ANOVA comparisons). Bars indicate ±SEM. **(C)** IgA and IgG clonotypes from six young and six old donors at different days were grouped by *IGHV* family (*x*-axis) and *IGHJ* (*z*-axis) gene usage, and the average clone size (*y*-axis) for each group was calculated. **(D)** IgA and IgG clonotypes from six young (Y) and six old (O) donors at different days were grouped by individual *IGHV* gene usage, and the average clone size for each group was calculated. The fold change of the average clone size at D0 from D7 (D7/D0) and at D28 from D7 (D28/D7) was calculated and is shown by different colors (grey as unchanged, green as fold decrease and red as fold increase). Serum levels of IgA antibodies specific for seven serotypes combined were determined using ELISA. Pearson correlation analysis was used to test the correlative relationship of age (*x*-axis in years) with changes in **(E)** serum anti-PPS IgA antibodies (*n* = 66) and **(F)** the average clone size (D7-D0, *n* = 12), *r*^2^ and *p* values indicated. The Goodness-of-Fit (solid lines) was analyzed by linear regression with 95% CI indicated (dashed lines).

It has been shown that clonal expansion after challenge is delayed and persists longer in old mice (Szabo et al., 2004). More recently, Lindner et al. (2012) reported that the intestinal IgA repertoire has fewer large, expanded, clones in old mice. In humans, we have previously shown vaccine-induced changes in CDR-H3 size and hydrophobicity in expanded clones of young people, but the changes are less obvious in the older group (Ademokun et al., 2011). Here we aim to investigate age and challenge-related changes in *IGH* gene usage. To ensure that our comparative analyses of clonal expansion reflect the effects of *in vivo* clonal expansion, rather than *in vitro* amplification from PCR, the *IGH* sequences were produced using the same number of cells in each sample. In the overall repertoire, large clones containing up to 687 member sequences appear at D7 and are seen less frequently at D28 in the young group, whereas in the older group the largest clone that appears at D7 has only 242 sequence members and some large clones are also observed at D28 (Figure 2A). Intraclonal variations in *IGHV* sequences indicate that these large clones likely represent *in vivo* expansion (Figure 6). To understand how clonal expansion occurs in different classes of B cells, *IGH* sequences were grouped by isotype for further analyses of clone size (Figures 2B–D). Significant increases in the average clone size at D7, as result of increased proportion of large clones, are seen in all isotypes in both age groups, although the increase in IgM clones is not as great as that seen in IgA and IgG clones. The pattern of vaccine responses in IgG and IgM clones, as indicated by changes in the average clone size, is similar between the two age groups (Figure 2B). The increase in average clone size was usually related to the accumulation of greater numbers of larger clone sizes, except in the case of IgA, where the young and old samples had comparable numbers of clones larger than 3, but the average clone size in young IgA clones at D7 is twice that of the old clones at D7 (Figure 2B; *p* < 0.005) This implies that the IgA clones in the young are larger than in the old. Consistent with this, the largest clones at D7 in the young in Figure 2A were IgA. Interestingly, the clone size is 2.5-fold larger in old IgA clones at D28 as compared with the young (Figure 2B; *p* < 0.005), and is larger than it was in the old at D7. So there may be a more delayed clonal expansion in IgA cells with age; while the young showed expansion at D7 and contraction back to D0 levels at D28, the level of expansion in the old may not yet have reached a peak by D28.

The vaccine response at D7 is very diverse at all ages in that changes in the average clone size are not restricted to particular *IGHV* family *IGHJ* gene combinations (Figure 2C). We calculated the fold difference in clone size between D7/D0 and D28/D7 for individual *IGHV* genes. At D7 there were 24 different *IGHV* genes of the IgA isotype and 16 different *IGHV* genes of the IgG isotype that had more than twofold increases in their average clone size (Figure 2D). The above age-related differences in the average clone size for IgA clones at D7 and D28 (Figure 2B) are a reflection of a diverse response, involving many different *IGHV* family *IGHJ* combinations and individual *IGHV* genes rather than any particular *IGH* genes (Figures 2C,D). The changes in the average clone size and serum anti-PPS IgA antibodies at D7 from D0 were also negatively correlated with age (Figures 2E,F).

### Age-related differences in CDR-H3 characteristics of expanded clones

**Figure 3:**
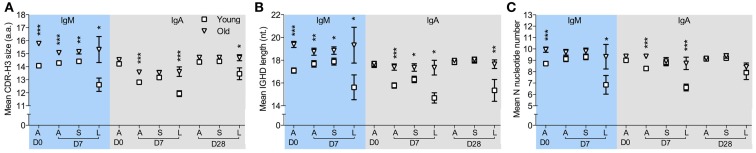
**Age-related changes in CDR-H3 characteristics from clonotypes in different sizes**. IMGT V-QUEST was used to determine the size of the CDR-H3 region and its components, i.e., N-nucleotide numbers and *IGHD* genes, in each *IGH* sequence. After clustered by CDR-H3 sequence motifs, IgM, and IgA clonotypes were sorted by clone sizes. **(A)** The mean size of CDR-H3 regions (in amino acids), **(B)** the mean length of overall *IGHD* genes (in nucleotides), and **(C)** the mean number of N-nucleotides from all clonotypes **(A)**, clonotypes ≤4 (S) and clonotypes ≥5 in size (L) were compared between six young (square) and six old (triangle) donors at D0, D7, and D28, using Mann–Whitney *U-*tests (*p < 0.05, **p < 0.005, and ***p < 0.0005). Error bars indicate ±SEM.

CDR-H3 regions have been regarded as indispensible Ig structures for antigen recognition, therefore alterations in the physiochemical properties of CDR-H3 peptides could potentially affect BCR binding ability and selection and expansion of antigen specific cells within a population may be detectable by changes in the CDR-H3 characteristics (Romero-Steiner et al., 1999; Kolibab et al., 2005b; Park and Nahm, 2011). We therefore compared CDR-H3 characteristics in *IGH* sequences from clones of different sizes at D7 after challenge. The clonotypes were divided into those seen four or less times in the sample (small clones) and those seen five or more times (large clones). The overall hydrophobicity and aliphatic index of CDR-H3 regions from large clones does not significantly differ between ages when all isotypes are considered together (data not shown). However, there are significant age-related changes in CDR-H3 size in IgA and IgM clonotypes from large clones (Figure 3A) that was not seen in IgG clonotypes (data not shown). IgM clonotypes show significantly larger CDR-H3 sizes in all clones regardless of clone size in the old samples than in the young at both D0 and D7 (Figure 3A). The differences in the CDR-H3 size are mainly due to an increase in the total length of *IGHD* genes (Figure 3B), not *IGHJ* (data not shown), and an increase in the numbers of N-nucleotides that reaches significance in expanded large clones (Figure 3C). There is also a significant age-related increase in the CDR-H3 size and CDR-H3 components in IgA clonotypes, although this is restricted to D7 and D28 post-challenge and is mainly in the larger clones (Figures 3A–C).

### Age- and challenge-related changes in hypermutation

**Figure 4:**
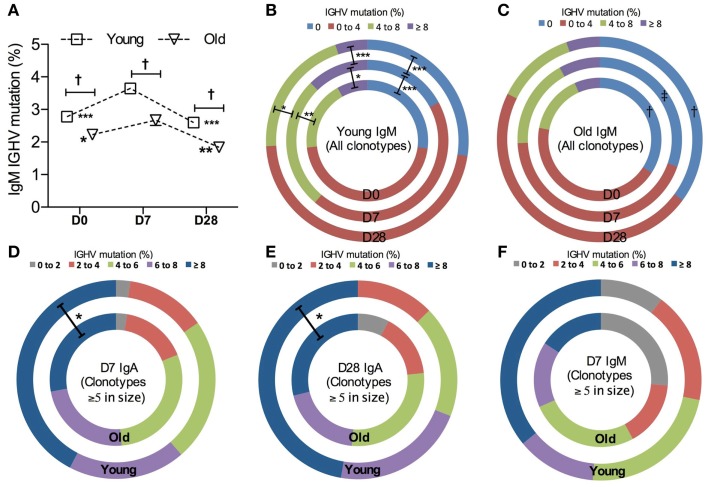
**Vaccine-induced changes in *IGHV* mutations**. **(A)** The frequency of *IGHV* mutations in each sequence was calculated to be the percentage difference from germline identity, determined by IMGT V-QUEST. Kruskal–Wallis comparisons were used to compare the mean *IGHV* mutation between IgM clonotypes from six young (squares) and six old (triangle) donors (age-related differences: ^‡^<0.0005) and between D0, D7, and D28 (temporal differences:*p < 0.05, **p < 0.005, and ***p < 0.0005, as compared with D7). Error bars indicate ±SEM. All IgM clonotypes were grouped by the level of *IGHV* mutation and the proportion of each group was compared using Chi-squared tests between days (D0: inner circles, D7: middle circles, and D28: outer circles; *p < 0.05, **p < 0.005, and ***p < 0.0005) and between six young **(B)** and six old **(C)** donors [^†^*p* < 0.05 and ^‡^*p* < 0.005, as indicated in **(C)**]. For clones ≥5 in size, IgM clonotypes at D7 **(D)**, IgA clonotypes at D7 **(E)**, and IgA clonotypes at D28 **(F)** were grouped by the level of *IGHV* mutations and the proportion of each group was compared between six young (outer circles) and six old (inner circles) donors, using Chi-squared tests (*p < 0.05).

Since the level of hypermutation accumulated in the Ig variable region often reflects the history of affinity maturation in B cell clones specific for a particular type of antigen, we compared *IGHV* mutation levels over the course of vaccination and in different age groups. The average mutation frequency is significantly increased in IgM clonotypes from both age groups at D7 after vaccination (Figure 4A), although the change at D7 from D0 in old IgM clones was threefold smaller than that in the young. Changes in overall *IGHV* mutation levels in IgM clones in response to vaccines and with age can also be demonstrated by the proportional alteration between unmutated versus heavily mutated IgM clones (Figures 4B,C).

A higher mutational frequency is observed with age in IgG clonotypes at all time points (*p* = 0.0003, Mann–Whitney *U*-test; data not shown). We did not see an increase in the overall mutation levels in IgA clonotypes after challenge in either age group (data not shown). Since IgA populations are reflective of prior challenge and hypermutation this perhaps would not be expected. However, looking solely at the large clones (five sequences or more per clone), presumably enriched for the ones responding to vaccination as opposed to those that were historically mutated, we saw that IgA clonotypes from old donors had a lower average frequency of mutation than the young (*p* = 0.0025, Mann–Whitney *U*-test). This may be due to the reduction in the proportion of heavily mutated, large IgA clonotypes with age (Figures 4D,E). A similar trend is also observed in IgM clonotypes (Figure 4F).

### Age and challenge-related changes in clonotypes and clonal expansion by *IGH* subclass

**Figure 5:**
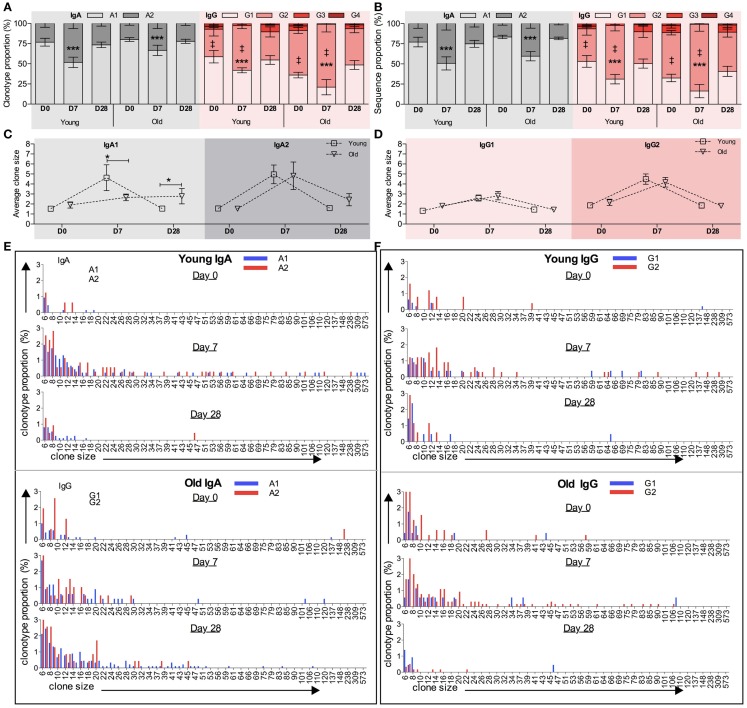
**Age and challenge-related changes in *IGH* subclass**. Internal motifs in the constant region were used to identify subclasses (IgA1, IgA2, IgG1, IgG2, IgG3, and IgG4) in each *IGH* sequence. The proportion (*y*-axis) of clonotypes clustered by CDR-H3 **(A)** and total sequences **(B)** of different IgA or IgG subclasses at D0, D7, and D28 was calculated for each donor individually before being collectively analyzed (temporal differences: ***p < 0.0005 using repeated measures ANOVA when compared with D7; age-related differences: ^‡^*p* < 0.005 using Mann–Whitney *U-*tests). Error bars indicate ±SEM. The average clone size (*y*-axis) of IgA1 and IgA2 clonotypes **(C)** and IgG1 and IgG2 clonotypes **(D)** was calculated to be the ratio of total sequences over total clonotypes for each donor individually, before being compared between young versus old ages (*p < 0.05; one-way ANOVA comparison). Bars indicate ±SEM. Clonotypes of **(E)** IgA subclasses (IgA1: blue bars and IgA2: red bars) and **(F)** IgG subclasses (IgG1: blue bars and IgG2: red bars) were grouped by their clone sizes (*x*-axis), and the proportion of each groups (*y*-axis) was calculated at D0, D7, and D28. Clones containing fewer than five sequences are not shown.

We previously reported that isotype-switched and innate-like IgM+ memory cells have distinct *IGHV* repertoires, and later that there are some similarities between the innate-like IgM+ memory cell repertoire and that of IgG2 and (to a lesser extent) IgA2 memory cells. Therefore we proposed that a large proportion of IgG2 and IgM (and possibly IgA2) memory cells may be subject to a different selection process from that imposed on other classes and subclasses of memory cells (Wu et al., 2010, 2011). In order to compare the vaccine response in B cells of different subclasses we stratified the overall clonotype repertoire by subclass. This was done by searching for subclass-specific motifs upstream of the pan-IgA or pan-IgG primer sequences, so that any difference in subclass within a particular class is not due to a difference in primer binding efficiency. In line with previous reports (Wu et al., 2010, 2011), over-representation of *IGHV3* and under-representation of *IGHV1* genes are seen in IgM, IgG2, and IgA2 clonotypes in both age groups at all time points analyzed when compared to IgG1, IgG3, and IgA1 clonotypes (data not shown). Within the IgA and IgG compartments the proportions of the subclasses vary with challenge, with a significant increase in the proportion of IgA2 and IgG2 clonotypes being seen in both age groups at D7 compared to D0 (Figure 5A; *p* < 0.005, repeated measures ANOVA). Although we failed to detect any age-related differences in Ig gene usage in IgA and IgG subclasses (data not shown), there is a significant difference in IgG subclass distribution with age at both D0 and D7, with the older group having an increased proportion of IgG2 compared with the young (Figure 5A; *p* < 0.0005, Mann–Whitney *U*-test). The increased proportion of IgA2 and IgG2 subclasses after vaccination are also observed when clone relatives (i.e., all sequences not just clonotypes) are included for analysis (Figure 5B).

Generally the differences in clonotype subclass distribution (Figure 5A) are mirrored in sequence subclass distribution (Figure 5B). However, in IgG there appears to be a greater bias toward IgG2 in sequences compared to clonotypes, indicating that in the young the IgG2 clonotypes belong to larger clones. Differences in IgA and IgG average clone size at the different time points are shown in Figures 5C,E respectively, with details of the distribution of clones in the different subclasses in Figures 5D,F. Significant age-related differences in the average IgA clone size (Figure 2B) are seen at D7 and D28, and appear to be mainly due to the IgA1 subclass having a smaller clone size at D7 but bigger at D28 in the old as compared with the young (Figure 5C). Although the average clone size in IgA2 does not seem to change with age, more detailed analysis of IgA2 clone size distribution shows that the frequency of large clones, containing over 20 sequence members, is lower at D7 (*p* = 0.002; Chi-squared test) but higher at D28 in old repertoires, as compared with the young (Figure 5E). In both age groups the average clone size of IgG2 is significantly bigger than IgG1 at D7 (*p* = 0.0008, paired *t*-test; Figure 5D). Thus, in addition to there being an increase in the number of different IgG2 clonotypes in the sampled repertoire at D7, the individual IgG2 clonotypes are expanded more than IgG1 clonotypes. The age-related increase in the proportion of IgG2 clonotypes at D7 and D28 (Figure 5A) is not accompanied by any significant difference in the clone size (Figure 5D). So although the older repertoire has a greater representation of IgG2 sequences these are not expanded any more than in the younger group. The IgG1 and IgG2 clone distribution is illustrated in more detail in Figure 5F.

### Clonal expansion as a result of recall immune responses

**Figure 6:**
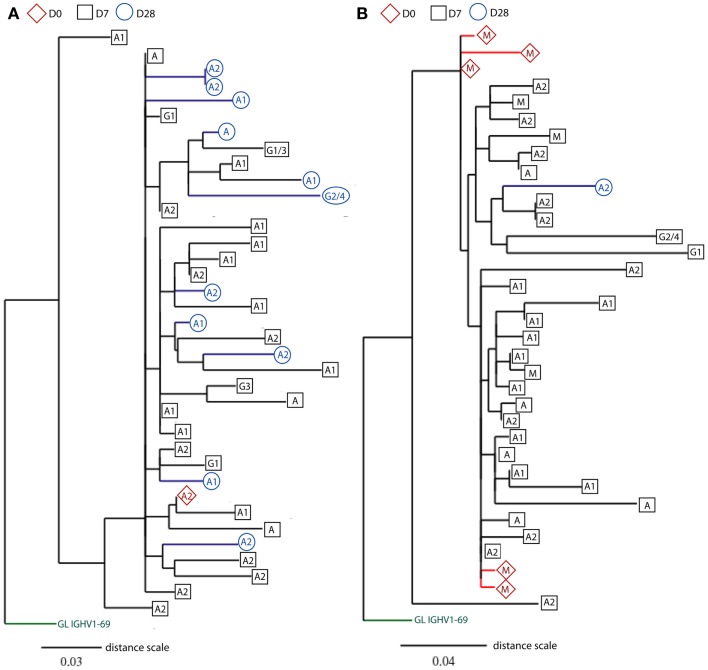
**Rooted phylograms show clonally related *IGH* sequences sampled at different days**. CDR-H3 sequence motifs were used to identify clone relatives of *IGH* sequences found across D0, D7, and D28. After editing and aligning *IGH* sequences with germline gene using DNAstar software, mutational phylogenetic trees were generated using the online Phylogeny Analysis program (Dereeper et al., 2010). Leaves represent *IGH* sequences sampled at different days (D0: red diamonds, D7: black squares, and D28: blue circles), and the putative germline (GL) genes are indicated at the base of trees in green. The class (IgG, IgA, and IgM) or subclass (indicated by numbers) of *IGH* genes is indicated in the shapes. Internal nodes represent hypothetical intermediates of *IGH* sequences, and the branch lengths indicate percentage mutation differences between *IGH* sequences in proportion to the distance scale. Only two illustrations of phylogenetic trees are shown**(A)** from an old donor aged 71 years and **(B)** from a young donor aged 22 years. For space consideration, sequences in the two clones are randomly chosen for illustration.

Previous studies show that influenza-specific cells (Wrammert et al., 2008) and anti-PPS plasma cells (Baxendale et al., 2010) can be detected in the serum prior to vaccination and their frequencies are increased at D7 following vaccination. Although our sequences were produced using unsorted PBMCs, large sequence numbers allowed us to track responding clones sampled at D7 back to their clone relatives sampled at D0 prior to vaccination, using mutational phylogeny analysis (Dereeper et al., 2010). We find a total of 648 different clonotypes (3.6% of all clonotypes) containing clonally related *IGH* sequences that represent cells sampled at different days (Table 2). 71% of these clones are of a single isotype, being significantly more frequent than those having *IGHM* sequences related to *IGHG* and *IGHA* sequences (12.8%; Chi-squared test, *p* < 0.0001). As expected, the proportion of clonotypes that share sequences across different time points increases to 20%, when only large clones are considered (Table 2). Out of these larger clones, 57 clonotypes (4.6%) contain *IGH* sequences that have already switched to IgA and IgG isotypes at D0 prior to vaccination (Figure 6A), suggesting a recall immune response by pre-existing memory B cells. Similarly, there are also clones that contain mutated IgM+ sequences at D0 (Figure 6B). Mutational phylogenetic trees show great diversification within expanded clones. Interestingly we also observe that sequences sampled at D0 do not always appear less mutated than those at D7 and D28. Similarly, *IGHM* sequences do not always appear before *IGHA*/*IGHG* sequences in the lineage tree. These observations have important consequences for future interpretation of data based on analysis of phylogenetic trees.

**Table 2:** **Numbers of clones with clonality between *IGH* sequences sampled at different days^1^**.

All clonotypes^2^										
Day**^1^**	Old (*n* = 7926^3^; 6 donors)					Young (*n* = 9774^3^; 6 donors)				
ISO^5^	AG	AGM	AM	GM	Single**^6^**	AG	AGM	AM	GM	Single
0 and 28	3	1	1	1	94	17	0	3	7	94
0 and 7	4	3	7	4	47	8	3	20	1	38
0 and 7 and 28	4	1	2	0	16	5	3	2	0	4
7 and 28	23	4	3	3	93	38	6	8	0	77

**Clonotypes ≥5 in size**										
**Day**	**Old (*n* = 671**^4^; **6 donors)**					**Young (*n* = 572**^4^; **6 donors)**				
**ISO**	**AG**	**AGM**	**AM**	**GM**	**Single**	**AG**	**AGM**	**AM**	**GM**	**Single**

0 and 28			1	1	25	11	0	0	1	22
0 and 7	3	3	1	1	19	5	2	13	1	9
0 and 7 and 28	4	1	1	0	12	4	3	1	0	2
7 and 28	20	4	2	0	36	32	6	3	0	24

*^1^CDR-H3 sequence motifs are used to identify clones containing *IGH* sequences that that have the same VDJ rearrangement but are sampled at Day 0, 7, and 28*.

*^2^All clonotype refers to all clones in various sizes*.

*^3^Numbers refers to all clonotypes produced by high-throughput sequencing*.

*^4^Numbers refer to clonotypes ≥5 in size produced by high-throughput sequencing*.

*^5^Iso refers to clones containing *IGH* sequences sharing the same CDR-H3 region but are of different isotypes (A for IgA, G for IgG, and M for IgM)*.

*^6^Single refer to clones containing IGH sequences of only one isotype*.

## Discussion

Improving the immunogenicity of vaccines against *S. pneumoniae* and influenza in the older person is a challenge (Artz et al., 2003; Hannoun et al., 2004). Effective antibody production is impaired in older people but the exact causes of this impairment have not been fully elucidated. An earlier study showed that affinity selection in the germinal center may be altered with age, this may be partly due to lack of appropriate help, for example from T cells, as well as intrinsic differences in the B cell affecting its ability to express AID (Banerjee et al., 2002; Frasca et al., 2008). We later showed that B cell diversity decreases with age and is associated with poor health (Gibson et al., 2009). Kolibab et al. (2005a) also suggest that changes in Ig gene usage may account for the different affinity of vaccine-specific antibodies between young and older populations. Thus there may be a causal relationship between the distortion of B cell repertoires and vaccine hypo-responsiveness with age. However, previous repertoire analyses to investigate similar subjects are restricted to certain genes and isotypes due to the small numbers of sequences available (Kolibab et al., 2005a; Smithson et al., 2005).

We have performed high-throughput sequence analysis of *IGH* repertoires in human peripheral blood in order to investigate B cell responses following vaccination with the pneumococcal and influenza vaccines. In contrast to the oligoclonal response previously reported in response to the pneumococcal vaccine (Zhou et al., 2004; Kolibab et al., 2005a), here we report a very diverse and resilient vaccine response, with increased clone sizes at D7 and normal repertoire resumed at D28 (Figures 2 and 5). The discrepancy in diversity with previous reports may reflect the number of sequences analyzed and/or the fact that the influenza vaccine was included in this study in addition to the pneumococcal vaccine. Since the peak of the plasmablast response is generally between days 6 and 8 (Cox et al., 1994; Wrammert et al., 2008), and plasma cells and plasmablasts have many more copies of immunoglobulin RNA per cell than B cells (Kuo et al., 2007), it is reasonable to assume that the clonal expansions seen at D7 represent the cells that are responding to vaccine. We can also see vaccine-induced changes in the CDR-H3 repertoire despite the fact that there are a large number of different antigens in this challenge. We previously showed that memory B cells in general have shorter CDR-H3 regions that are more hydrophilic (Wu et al., 2010), implying that Ig genes with these characteristics are preferentially selected in many different responses. These changes are also seen at D7 here (Figures 1E,F) and these data together strongly imply that the antibody immune response to challenge is skewed toward antibodies with certain characteristics of the antigen binding region even though the antigens themselves can be quite variable.

Many studies use specific serum IgG levels as a correlate of vaccine protection, although this metric is not always the best indicator. There are no age-related differences in anti-PPS IgG serology, but there is a significant age-related decrease in serum antibody function as determined by the opsonophagocytic assay (Anttila et al., 1999). Recent evidence suggests that this is due to a decrease in IgM antibody, since depletion of IgM from serum results in decreased opsonophagocytic activity (Park and Nahm, 2011; Sasaki et al., 2011). Our repertoire analysis does not show any age-related changes in IgG, except for a higher mutational frequency in the older group that is likely due to longer prior exposures to challenge with age. Nor do we see any significant age-related differences in the level of clonal expansion of IgM sequences. We do, however, see significant age-related differences in the CDR-H3 characteristics (Figure 4) and levels of mutation in the IgM repertoire (Figure 3), with generally less mutation and longer CDR-H3 in the old (both these factors being more characteristic of naïve B cell repertoires rather than memory B cell repertoires). The differences were significant at all time points so may not necessarily be confined to a change in the response. The IgM repertoire includes the naïve B cell population as well as IgM memory cells, so a change in the proportions of these two populations would also have an effect on our repertoire observations; thus, a decrease in IgM memory cells such as has been previously suggested may account for some difference (Shi et al., 2005). However, both age groups did show an increased level of mutation at D7 and a decrease at D28, although to a lesser extent in the old group. In order to look at the differences between age groups in the response without the background of naïve cells we split the data to look at sequences that were part of large clones in isolation, on the assumption that if a clone has expanded it is part of the IgM memory response rather than the naïve B cells in the background. The older IgM response repertoire had longer CDR-H3 and less mutation (Figures 3 and 4), which would strongly suggest that there is an age-related defect in the normal mechanisms of selection and hypermutation in IgM memory. We do not know what the specificity of these expanded IgM clones is, although based on previous literature we would hypothesize that they are T-independent responders to the polysaccharide antigen (Lortan et al., 1992). IgG2 antibodies are also thought to respond to T-independent polysaccharide antigens (Lortan et al., 1992) so it is interesting that alongside the defect in IgM memory repertoire there is a skewing in favor of IgG2 use in the IgG repertoire of older people.

A role for IgA in the protective vaccine response against influenza and pneumonia has not previously been highlighted, and the removal of IgA from serum was not shown to have any effect on the opsonophagocytic capability of post-vaccine serum. Since these respiratory diseases originate at mucosal surfaces it would seem plausible that IgA has some vital function, even if not in the circulation. It is clear from our data that there are significant differences in the IgA response in older people. In a similar manner to the IgM memory cells there is less hypermutation in the expanded clones and they have larger CDR-H3 regions. The degree of clonal expansion is less overall and takes much longer, to the extent that is still appears to be occurring at D28 after the younger group has contracted the response. The IgA serum antibody response is short lived. In contrast to IgG and IgM antibodies, which increase in serum with maximum values at D28, the maximal serum level of IgA is at D7 in the normal young population and it decreases by D28 (Ademokun et al., 2011). The clonal expansions of all three isotypes peaks at D7 and contracts at D28 in the blood (Figure 2B). Thus for IgG and IgM one can envisage a scenario where cells have left the blood to reside in a niche where they continue to secrete antibody into the circulation. However the serum IgA antibody concentration seems to mirror the clonal expansion data, which may indicate that the cells are short lived and do not go on to secrete antibody in survival niches, or perhaps they do survive but secrete IgA antibody at mucosal surfaces rather than into the circulation.

Since the most striking age-related difference in these data was in the IgA response we split the data into IgA1 and IgA2 sequences. IgA1 has previously been associated with serum responses and IgA2 with mucosal responses (Russell et al., 1992). It is clear from our lineage tree analysis that IgA1 and IgA2 can be quite closely related, since we find many clones containing both subclasses (Figure 6). However in Figure 5C we see that the main age-related difference in clonal expansions that we saw at D7 and D28 is mainly due to IgA1 rather than IgA2, and we also find clones which do not mix the subclasses, so it is possible that certain types of antigens/responses may elicit one subclass only.

The existence of many clones with relatives in both pre- and post-vaccination samples (Table 2) indicates that some of these clones may be very large even before vaccination. The chances of finding a particular clonotype in a sample are dependent on the number of cells sampled, the total number of cells in the blood, and the total number of clone members in the blood. We would not expect to find a particular clonotype more than once if it were not part of an expanded clone. In the simplest terms, if we assume that there are 10^8^ B cells in the blood, and we have found two related sequences in a sample of 5000, then there could be approximately (10^8^/5000)^2^, or 40,000, related sequences in that one clone in the blood altogether. Also, if we assume that an expansion at D7 originated from a single unique precursor in the blood at D0, the chances of sampling and sequencing that precursor would be 1 in 10^8^. Since we see nearly 400 examples of clones where there are related sequences at D0 then we can provisionally conclude that many expanded precursors of cells with specificity for these vaccines are already present in the blood pre-challenge. Whether these pre-existing specificities are mono-specific for the antigens in the vaccine or are cross-reactive from a prior, related, challenge cannot be determined since it was impossible to determine the prior extent of exposure to influenza or *S. pneumoniae* in the participants. These high-throughput data have also shown that we need to be careful about interpretation of lineage trees with respect to inference of chronological ordering since the trees can be quite complex (Figure 6). If there has been extensive prior expansion of cells in the blood it cannot be assumed that all clones in the expansion will have mutated at the same rate or in the same reaction. Hence a random sampling will not always result in the samples from the earlier time points appearing at the top of the lineage trees (i.e., with less mutations from germline). Similarly, extensive expansion followed by random switching and sampling may result in a seemingly impossible succession of switching events, such as *IGHM* sequences appearing downstream of *IGHA* sequences. However this could simply mean that a cell has expanded without mutation, 50% of them have switched and the sampling has picked up one of the switched parents together with offspring of one of the unswitched parents.

In conclusion, our high-throughput *IGH* repertoire analyses have demonstrated that we can visualize an immune response to vaccine by the expansion of clonotypes expressing particular Ig genes, and with particular CDR-H3 characteristics. The large clonal expansions indicate a complex recall response. There are significant age-related differences in the response with respect to subclass distribution, particularly in the extent and timing of IgA clonal expansion and skewing toward greater use of IgG2. Older responding IgM and IgA clonotypes are also less mutated and use a longer CDR-H3, which might affect antigen recognition. Although much more now needs to be done to explore the significance of the age-related changes in IgA responses described here, our work does highlight the critical need to consider different classes and subclasses of antibody in vaccine studies in general.

## Author Contribution

Yu-Chang Bryan Wu designed and carried out experiments, analyzed data and wrote the manuscript; David Kipling designed and ran the data handling and analysis scripts, analyzed data, and wrote the manuscript; Deborah K. Dunn-Walters, oversaw the project, designed experiments and analytical tools, carried out data analysis and wrote the manuscript.

## Conflict of Interest Statement

The authors declare that the research was conducted in the absence of any commercial or financial relationships that could be construed as a potential conflict of interest.
